# Freeze-Drying Microencapsulation of Hop Extract: Effect of Carrier Composition on Physical, Techno-Functional, and Stability Properties

**DOI:** 10.3390/antiox12020442

**Published:** 2023-02-10

**Authors:** Simona Tatasciore, Veronica Santarelli, Lilia Neri, Rodrigo González Ortega, Marco Faieta, Carla Daniela Di Mattia, Alessandro Di Michele, Paola Pittia

**Affiliations:** 1 Department of Bioscience and Technologies for Food Agriculture and Environment, University of Teramo, Via Renato Balzarini 1, 64100 Teramo, Italy; 2 Faculty of Science and Technology, University of Bolzano, Piazza Università, 39100 Bolzano, Italy; 3 Department of Physics and Geology, University of Perugia, Via Pascoli, 06123 Perugia, Italy

**Keywords:** hop extract, bioactive compounds, microencapsulation, freeze-drying, gum arabic, maltodextrin

## Abstract

In this study, freeze-drying microencapsulation was proposed as a technology for the production of powdered hop extracts with high stability intended as additives/ingredients in innovative formulated food products. The effects of different carriers (maltodextrin, Arabic gum, and their mixture in 1:1 *w*/*w* ratio) on the physical and techno-functional properties, bitter acids content, yield and polyphenols encapsulation efficiency of the powders were assessed. Additionally, the powders’ stability was evaluated for 35 days at different temperatures and compared with that of non-encapsulated extract. Coating materials influenced the moisture content, water activity, colour, flowability, microstructure, and water sorption behaviour of the microencapsulates, but not their solubility. Among the different carriers, maltodextrin showed the lowest polyphenol load yield and bitter acid content after processing but the highest encapsulation efficiency and protection of hop extracts’ antioxidant compounds during storage. Irrespective of the encapsulating agent, microencapsulation did not hinder the loss of bitter acids during storage. The results of this study demonstrate the feasibility of freeze-drying encapsulation in the development of functional ingredients, offering new perspectives for hop applications in the food and non-food sectors.

## 1. Introduction

In food production, the use of plant extracts with functional properties and technological functionalities represents a new strategy to replace synthetic additives or ingredients by responding to the growing request of consumers and stakeholders, especially in the organic sector, for innovative, sustainable, clean label, healthy, and high-quality food products. In this context, hop cones, inflorescences of the female plant of *Humulus lupulus* L. have attracted the attention of researchers and food industries for their high content of secondary metabolites, i.e., bitter acids, essential oils, and polyphenols, which besides flavouring, bittering, and stabilizing properties show different biological activities [1] and technological functionalities (antioxidant and antibacterial activity). Hops’ bitter acids are secreted by lupulin glands and consist of two related series of homologues, i.e., α-acids or humulones and β-acids or lupulones, respectively, present in large and minor amounts. The α-acids consist of a mixture of six humulone analogues, among which the major ones are humulone (35–70% of total α-bitter acids), cohumulone (20–65% of total α-bitter acids), and adhumulone (10–15% of total α-bitter acids), while β-bitter acids consist of lupulone (30–55% of total β-bitter acids), colupulone (20–55% of total β-bitter acids), adlupulone (5–10% of total β-bitter acids), pre-lupulone, and post-lupulone [2,3]. The antimicrobial activity of these compounds is well-documented and concerns mostly Gram-positive species, such as *Lactobacillus, Streptococcus, Staphylococcus, Micrococcus,* and *Bacillus*, and certain fungi, such as *Penicillium* and *Aspergillus* species [4,5]. Furthermore, hop α- and β-acids possess significant antioxidant activities in vitro, which offers the possibility of using hop extracts as natural antioxidants [6]. In particular, non-oxidized hop bitter acids (humulones and lupulones) and hop extracts rich in these compounds have shown strong quenching activity against DPPH hydroxyl and peroxynitrite radicals and inhibition of lipid peroxidation in the thiobarbituric acid (TBA) assay and oxidation of β-carotene and linoleic acid. The measured antioxidant potential of α-acids was usually slightly higher than that of β-acids, while derivatives of both (e.g., iso-acids) were found to have a much lower effect. The β-triketone moiety was proposed as the bioactive site contributing to the antiradical activity of hops-derived bitter acids [7].

Hop polyphenols, which depending on the variety, harvest time, and year of production can represent also up to 14% of the hops’ dry matter, comprise flavan-3-ols, flavonols, phenolic carboxylic acids (derivatives of benzoic acid and cinnamic acid), and prenylflavonoids [5,8,9]. These compounds are not essential in the brewing process but play an important role in beer quality contributing to the colour, astringency, and stability of the beverage. Moreover, several studies have shown that hop polyphenols, besides their antioxidant activity, exert a strong protective action on human health thanks to their immune-modulatory actions, and anti-inflammatory, anticancer-related, and antibacterial activity [10,11]. In particular, xanthohumol and other prenylflavonoids have health-promoting, protective, and preventive effects against many civilization-related diseases (e.g., cancer, osteoporosis), exert anti-infective effects against Gram-positive bacteria (e.g., *S. aureus*, *S. mutans*) and viruses, and the effects are enhanced in hop extracts, where they are combined with iso-α-acids [12]. 

The recovery of polyphenols and bitter acids from hop cones through food-grade solvents requires liquid extraction processes that have been recently investigated in previous studies [2,13]. However, the direct use of liquid extracts in food formulations can find limited applications [14]. Furthermore, these compounds are sensitive to environmental stresses, and exposure to high temperatures, oxygen, water, and light during processing, storage, and transport can determine the loss of their biological value, bioavailability, solubility, and functionality [15]. Lastly, many of these molecules have a very low water solubility and a strong astringent and bitter taste, which hinders their direct use in food products.

A common technique applied to overcome these drawbacks is microencapsulation by desolvation (e.g., spray drying) since it allows the production from liquid food-grade plant extracts of powdered ingredients with easy to handle, dose, and deliver into food products with improved technological functionalities and stability. Furthermore, microencapsulation may help mask unpleasant tastes and control the release of the core components. However, the encapsulation efficiency, functionalities, and stability of microencapsulated extracts are strictly dependent on the type of wall material chosen for the encapsulation process. Among the wall materials used for microencapsulation, different classes of compounds could be used (e.g., proteins, polysaccharides, hydrocolloids), either singly or in combination to leverage the synergistic effect of their technological functionalities for an improved encapsulation performance [16,17,18].

Many studies related to microencapsulation of fruit and plant extracts using several techniques and wall materials are reported in the literature [19], but only one focused on hop extract [20]. Therefore, this study aimed to use freeze-drying microencapsulation for the production of hop extract powders as functional additives/ingredients and to evaluate the effect of different carriers on their quality and stability properties. Freeze-drying was applied as a microencapsulation technique due to the low process temperature, which is particularly suitable for the entrapment and protection of sensitive hop bioactive compounds such as bitter acids and polyphenols [21]. Maltodextrin and gum Arabic were chosen as coating materials based on their good glass-forming properties and their wide application as carriers for the encapsulation and protection of plant extract bioactive compounds for food purposes [19,20,22]. 

## 2. Materials and Methods

### 2.1. Materials

A single batch (2 kg) of hop cones (*Humulus lupulus*, L.) cv. Herkules was purchased from a local producer in the Hallertau region (Germany) and used for all the experiments. Freshly harvested cones were dried in traditional kiln driers. Fresh hop cones were put in three layers in a continuous process at 65 °C until moisture content less than 12% was obtained, and then were packed under a vacuum in high-barrier plastic bags and stored at −40 °C until use. Maltodextrin (MD) Dextrose Equivalent (DE) 7.5–9.9 was purchased from Cargill S.r.l. (Milan, Italy) and Arabic gum (GA) from Kerry Ingredients & Flavours Ltd. Global Technology & Innovation Centre (Millennium Park Naas, Kildare, Ireland). All the reagents for analysis were purchased from Sigma-Aldrich (Steinheim, Germany). 

### 2.2. Samples Preparation

The procedure for the preparation of the samples is briefly described in the scheme reported in Figure S1 of the Supplementary Materials.

#### 2.2.1. Production of Concentrated Hop Ethanolic Extract (ExC)

The extraction conditions (i.e., method and time) to obtain the hop ethanolic extract were chosen on the basis of a previous study [13]. Briefly, after grinding and sieving, the dried hop powder was added (matrix:solvent ratio of 1:50 *w*/*v*) to ethanol: water 50:50 (*v*/*v*) and treated with ultrasound (100 W, 50 kHz) for 30 min by an ultrasound bath LAB SONIC LBS 1 (Falc Instruments, Treviglio, Bergamo, Italy). Thus, the sample was centrifuged at 2470× *g* for 10 min at 4 °C, and the collected supernatant was filtered under vacuum to obtain the hop ethanolic extract. Finally, a solvent-free hop extract (ExC) was obtained by concentration on a rotary evaporator (Buchi R-100) at 45 °C.

#### 2.2.2. Freeze-Drying Microencapsulation of Hop Extract

Before freeze-drying microencapsulation, the ExC was resuspended in a 0.02% *w*/*w* Tween 20 solution using a 1:66 *w/v* ExC: Tween 20 solution ratio. Thus, the 12% (*w*/*v*) of coating material, i.e., Maltodextrin (MD), Arabic gum (GA), and a mixture 1:1 (*w*/*w*) of maltodextrin and Arabic gum (MD-GA), was added to the resuspended ExC and dissolved by mixing under continuous stirring (400 rpm) at 35 °C for 30 min. In parallel, three different samples without ExC were prepared by the dissolution of each coating material in 0.02% *w*/*w* Tween 20 solution and used as controls.

Freeze-drying microencapsulation was performed using a Labogene (Allerød, Denmark) Scanvac Coolsafe freeze-dryer. Each sample was put in Petri dishes and frozen in a freezer (Nuve, FR 490) at −40 °C for 24 h. The freeze-drying process was carried out at 0.316 hPa, increasing the temperature of the shelves from −40 °C to 17 °C in 24 h. 

After freeze-drying, the hop extracts encapsulated with maltodextrin (ExMD), Arabic gum (ExGA), and with a combination of the two carriers (ExMD-GA), and the corresponding control samples (MD, GA, MD-GA) were grounded, sieved (ISO 3310–ISO 565–NF X 11-504–ASTM E11, w: 500 µm) in a glove box system under a nitrogen atmosphere, and stored at −40 °C before physio-chemical analysis.

### 2.3. Storage Stability Tests

Stability tests were carried out on encapsulated hop extracts (ExMD, ExGA, ExMD-GA) packaged in glass vials under nitrogen and stored at different temperatures (5, 20, 35, 50 °C) for different times (0, 7, 14, 21, 35 days). To evaluate the effect of microencapsulation and of the coating materials on the stability of hop bioactive sensitive compounds, the ethanolic extract (ExC) was also packed and stored in glass flasks at 50 °C. After storage, both the encapsulated samples and ExC samples were characterized for the total phenolic content (TPC), ferric reducing antioxidant power (FRAP), 2,2-azinobis (3-ethyl-benzothiazoline-6-sulfonic acid) (ABTS^+^) radical scavenging activity, and the content of α-acids and β-acids, as described in the following sections.

### 2.4. Characterization of Concentrated Hop Extract (ExC)

#### 2.4.1. Total Phenolic Content (TPC), Antioxidant Capacity by ABTS and FRAP Assays

Total phenolic content (TPC) was determined using the Folin–Ciocalteu reagent and the ABTS radical cation discolouration assay as described by Santarelli et al. [13]. The results of the assays were expressed, respectively, as mg gallic acid equivalent (GAE) per g of ExC and μmoles of Trolox equivalent per g of ExC. Since ExC represents a solvent-free extract data can be considered as expressed as on dry matter (dm).

Ferric reducing antioxidant power was determined according to the method described by [23], with slight modifications. Briefly, 200 μL of solution opportunely diluted was mixed with 1.3 mL of the FRAP reagent obtained by mixing acetate buffer (300 mM, pH 3.6), and 10 mM TPTZ (2,4,6-tripyridyl-s-triazine) solubilized in HCl 40 mM and FeCl_3_ 20 mM, in the ratio 10:1:1. The solution was vortexed and incubated at 37 °C for 30 min. The absorbance was measured at 593 nm, and FeSO_4_·7H_2_O standard solutions were used to calibrate the method. Results were expressed as μmol Fe^2+^Eq g^−1^ of dry matter. TPC, ABTS, and FRAP were determined after dissolution (1:50 *w*/*v*) of the ExC in a mixture (50:42:8) of ethanol:water:acetic acid [24]. The results were the average of three independent measurements.

#### 2.4.2. α-Acids and β-Acids Assay

The determination of α-acid and β-acid content was performed according to the official ASBC Hops-6 method (American Society of Brewing Chemists, ASBC, 2012). α- and β-acid content was determined by different spectrophotometric lectures at 275, 325, and 355 nm. The results were the average of three independent measurements, and data were expressed as % *w*/*w*.

### 2.5. Physical and Physico-Chemical Characterization of Encapsulated Hop Extracts

#### 2.5.1. Water Activity, Moisture Content, and Solubility

Water activity (a_w_) was measured at 25 °C using the hygrometer Aqua Lab 4TE (Aqua Lab, Decagon Devices Inc, Pullman, WA, USA). Moisture content was determined gravimetrically by oven drying at 105 °C until constant weight. 

Solubility was determined according to a previous study [25]. Briefly, one g of the sample was mixed with 100 mL of distilled water, and the mixture was stirred in a magnetic stirrer for 30 min. The solution was then centrifuged at 3000× *g* for 5 min. An aliquot of 4 mL was oven-dried at 105 °C for 24 h. The solubility was calculated by weight difference and expressed in a percentage. All determination was performed in triplicate.

#### 2.5.2. Flow Properties

The bulk density, tapped density, flowability, and cohesiveness expressed in terms of Carr’s index (CI) and the Hausner ratio (HR) of differently encapsulated hop extracts were determined according to [26]. All determination was performed in triplicate.

#### 2.5.3. Colour and Colouring Power

Colour analysis was performed using a Konica Minolta Chroma Meter CR-5 spectrophotocolorimeter (Konica Minolta, Osaka, Japan) as reported by Santarelli et al [27] with slight modifications. Briefly, the measures were carried out on the encapsulated samples using a target mask with a measured area of 8 mm. Colour was assessed in CIELab coordinates; the hue angle (h°) was calculated as h° = tan^−1^ (b*/a*). All determinations were performed in triplicate.

The colouring power of the powders was determined using a Konica Minolta Chroma Meter CR-5 spectrophotocolorimeter (Konica Minolta, Osaka, Japan) in transmittance mode by evaluating the CIELab coordinates of the soluble fraction (supernatant) [28] obtained by resuspending the hop powders in distilled water (1%, *w*/*v*) stirring for 30 min, and centrifugation at 1000 rpm for 10 min. The hue angle (h°) was calculated as h° = tan^−1^ (b*/a*). All determinations were performed in triplicate.

#### 2.5.4. Sorption Isotherm 

Water vapour sorption isotherms were determined using the microclimate method. The equilibrium relative humidity of airtight jars was fixed with salt solutions characterized by a_w_ values in the range of 0.11–0.85. Jars were stored in the dark, at room temperature (25 °C). Analyses were carried out in triplicate, on encapsulated powders’ aliquots of approximately 0.1 g preliminarily dried under P_2_O_5_. Each sample was periodically weighed to ensure equilibrium was reached. 

Water sorption isotherms were modelled by fitting the data to the Guggenheim–Anderson–de Boer (GAB) equation reported below (Equation (1)):

(1) $$\frac{m}{m_{0}}=\frac{C\;K\;a_{w}}{\left(1-Ka_{w}\right)\left(1-K\;a_{w}+C\;K\;a_{w}\right)}$$

where *m* is moisture content (d.b.); *a_w_* is the water activity; and $m_{0}$, *K*, and *C* are the three free sorption parameters characterizing sorption properties of the material. The $m_{0}$ denotes moisture content corresponding to the ‘monomolecular layer’ on the whole free surface of the material; the parameters *K* and *C* depend on temperature by Arrhenius-type equations with corresponding molar sorption enthalpies [29].

#### 2.5.5. Thermal Analysis

Thermal analysis of the powders was carried out by using a differential scanning calorimeter (DSC 8500, PerkinElmer, Waltham, MA, USA). Analysis was carried out to determine the thermal properties and in particular the glass transition temperature (Tg) of amorphous freeze-dried powders with and without the hop extract. Instrument calibration was performed using indium. An aliquot of the powder accurately weighed (ca. 5–7 mg) was placed into DSC aluminium pans (50 μL, PerkinElmer) and hermetically sealed with pierced aluminium lids to allow evaporation of residual water upon heating scan measurement. Samples were heated from 20 °C up to a maximum of 200 °C at 5 °C/min and cooled at 10 °C/min to the initial temperature. A second heating scan at 5 °C/min was used to determine the onset and change of specific heat at Tg (ΔCp) using the PyrisTM software (PerkinElmer). The maximum temperature in the thermal analysis cycle was set depending on the sample moisture content.

#### 2.5.6. Microstructure Analysis

The morphology of the samples was analyzed by field emission scanning electron microscopy FE SEM LEO 1525 (ZEISS). The sample was deposited on an aluminium support using conductive carbon adhesive tape. Before the analysis, the samples were metalized with a thin layer of chromium (8 nm). Measurements were carried out using an In-lens detector at 5 kV.

### 2.6. Chemical Characterization of Encapsulated Hop Extracts

Before each analysis, 0.180 g of each encapsulated extract was dissolved in 3 mL of distilled water. The complete solubilization of the powder was obtained using ultrasound (100 Watt, 50 kHz) for 3 min (ultrasonic bath LAB SONIC LBS1 3Lt 2015, Falc Instruments, Treviglio, Bergamo, Italy) and 30 s of vortexing. When necessary, the solubilized samples were stored at −40 °C until analysis, which occurred within one day.

#### 2.6.1. Total Phenolic Content and Antioxidant Capacity of Hop Powder by FRAP and ABTS Assays

Before analyses, the pre-solubilized samples were subjected to preliminary extraction procedures. Briefly, 250 μL of each sample was mixed with 250 μL of distilled water and 1 mL of extraction solvent (50:42:8 ethanol:water:acetic acid), vortexed for 30 s, and centrifugated for 10 min at 2500× *g* at 10 °C. The supernatant was recovered, opportunely diluted, and used for the TPC, FRAP, and ABTS assays. The first two assays were performed as described in Section 2.4.1., while the ABTS assay was carried out according to Sarabandi et al. [30].

#### 2.6.2. α-Acids and β-Acids Assay

The determination of α-acids and β-acids was performed on pre-solubilized samples according to the official ASBC Hops-6 method, (American Society of Brewing Chemists ASBC, 2012) as reported in Section 2.4.2. 

### 2.7. Encapsulation Efficiency and Yield of Total Phenolic Content

For the determination of surface phenolic content (SPC), the procedure described by Ravichai and Muangrat [24] was applied. A total of 10 mg of encapsulated hop powder was resuspended in 1 mL of ethanol:methanol (1:1), vortexed for 1 min, and centrifuged at 6000 rpm for 15 min. The total phenolic content of the SPC fraction was analyzed by the Folin–Ciocalteu assay as described in Section 2.4.1.

The encapsulation efficiency (TPC EE%) was calculated by applying the following equation (Equation (2)):

(2) $$EE\left(\%\right)=\frac{Total\;phenolic\;content\left(\frac{mg\;GAE}{g\;powder}\right)-Surface\;phenolic\;content\left(\frac{mg\;GAE}{g\;powder}\;\right)}{Total\;phenolic\;content\left(\frac{mgGAE}{g\;powder}\right)}\times 100$$

The load Yield (Y), indicating the amount of total phenolic content (TPC) still present in the micro-encapsulated matrix after the freeze-drying process, was computed by applying the following equation (Equation (3)):

(3) $$Y\%=\frac{Total\;phenolic\;content\;\left(\frac{mg\;GAE}{g\;powder}\right)}{Calculated\;value\;of\;added\;phenolic\left(\frac{mg\;GAE}{g\;dry\;hop\;extract}\right)\;}\times 100$$

### 2.8. Statistical Analysis

Data were reported as mean and standard deviation and additionally analyzed by one-way ANOVA using STATISTICA for Windows (StatSoftTM, Tulsa, OK, USA) software. Significant differences were calculated by the Tukey (HSD) test at a significance level *p* < 0.05.

## 3. Results

### 3.1. Characterization of Concentrated Hop Extract (ExC) 

In Table 1, α- and β-acids and total phenolic content, as well as the antioxidant activity of the concentrated hop extract (ExC) is reported. To make comparisons with other data reported in the literature, the results were also expressed on a dried hop cones powder basis.

Hop cones of the Herkules variety showed a total phenolic content of about 158 mg GAE g^−1^ dm. This value is higher than that determined by Santarelli et al. [13], in hop cones of Cascade, Magnum, and Marynka varieties extracted with ethanol 50%, by Liu et al. [6] in cones of SA-1, Tsingdao flower, Nugget, Chinook, and Marco Polo varieties extracted with pure ethanol, and by Lyu et al. [31] in Calypso, Cascade, Cluster, Magnum, and Saaz1 varieties. This result highlights the suitability of the Herkules cultivar for the production of hop extracts rich in phenolic compounds. 

As concerns the α-acids and β-acids, the content was about 41% and 8% (*w*/*w*), respectively. These values were higher than that reported by Wu et al. [32] for a concentrated hop extract obtained from commercial hop pellets by 55% hydroethanolic extraction. As regards the antioxidant activity, ExC showed a ferric reducing power of 1284 µmol Fe^2+^ g^−1^ dm and a TEAC of 757 µmol g^−1^ dm. The result of the FRAP and ABTS assay were, respectively, similar to and lower than those reported by Mafakheri et al. [33] and by Önder et al. [34] for concentrated wild hop extracts. Overall, comparing the total phenolic content and the antioxidant activity of hops with those of other categories of spices and foods generally considered to be rich sources of antioxidant compounds [35,36], it is possible to highlight that this officinal plant can be considered an extraordinary source of antioxidant compounds. 

### 3.2. Physicochemical Characterization of the Encapsulated Hop Extracts

#### 3.2.1. Water Content, Water Activity, and Solubility

In Table 2, the moisture content and the physico-chemical properties of the encapsulated hop extracts are reported. It can be observed that all the samples, after freeze-drying, are characterized by a very low water content with values ranging between 2 and 5%. Comparing the three samples, it can be highlighted that at equal freeze-drying conditions, the use of Arabic gum as the carrier material, compared to the sole maltodextrin, negatively affected the water removal, and led to powders with higher moisture content and, accordingly, also with higher a_w_ values.

All the powdered extracts presented the same water solubility (*p* > 0.05), which was high (ca. 99%) as expected since both maltodextrins (DE 7.5–9.9) and Arabic gum are characterized by high solubility in water [22] and constitute 88% of the hop powders. 

#### 3.2.2. Bulk Density, Tapped Density, Flowability, and Cohesiveness

The bulk density, tapped density, flowability, and cohesiveness data of the differently encapsulated hop extract are shown in Table 2. Irrespective of the type of encapsulating agent, all the powders showed low bulk and tapped density values, which is a common characteristic of freeze-dried microencapsulated extracts due to their irregular morphological structure, which forms hollow areas between the particles [37,38]. For bulk density, the highest value was observed for ExGA followed by ExMD and ExMD-GA with significative (*p* < 0.05) differences among the samples. This result agrees with the results reported by Mahdavee Khazaei et al. [38] in freeze-dried encapsulated saffron petal extract and could be related to both the different size distribution of the crushed particles and frangibility and flow properties of produced powders, and to the higher molecular weight of GA [39] than MD [40]. In fact, the higher is the weight of the powder, the easier it accommodates into the spaces between the particles, occupying less space and resulting in higher bulk density values [41]. A similar trend was evidenced for the tapped density with the highest value found for ExGA. The bulk and the tapped density are important properties of powdered ingredients. In particular, bulk density seems to have an impact on the powders’ stability. Some authors suggest that the larger the number of spaces between particles, the greater the amount of oxygen available to lead to degradation reactions [41,42]; however, Desobry et al. [43] suggest that higher bulk density means only that the particles could fit more compactly and has shown that it had no effect on oxidation rates. Tapped density is instead related to the transport, packaging, and marketing of powders since a high-density powder can be stored in a smaller container with respect to a lower-density product [44].

Flowability and cohesiveness were measured by the evaluation of the Carr index (CI) and the Houser ratio (HR), respectively. Based on the classifications reported by Jinapong et al. [45], all the encapsulated hop extracts were characterized by bad flowability and high cohesiveness with CI ranging between 35–45 and HR between 1.6 and 1.9. The rationale behind the powders’ poor flowability is the small particle sizes (<500 µm) and the large surface area per unit mass of powder, which affects the contact surface area between powder particles available for cohesive forces and frictional forces to resist flow [46].

#### 3.2.3. Colour and Colouring Properties 

The colour and colouring power of the powders expressed by the a*, b*, L*, and h° parameters are reported in Table 2. All the samples showed negative a* and positive b* values, indicating a tendency to a yellowish and greenish colour. However, due to the higher lightness (L*) and b* values, ExMD was brighter and more yellow compared to the other samples containing Arabic gum. Since all the samples were produced with an equal extract/carrier ratio, the differences among the samples are due to the different colourimetric properties of the carrier materials. In particular, the white colour of maltodextrin contributed to the lightening of the hop powders [47]. Moreover, the highest value of the hue angle, a parameter related to the perceived colour of the powders, was observed in ExMD, and no significant differences were detected between ExGA and ExMD-GA powders.

Likewise, the three encapsulated hop extracts are characterized by a different colouring power with samples containing maltodextrin showing L* and h° values slightly higher than samples containing only Arabic gum, i.e., a colour slightly brighter and with more tendency to green.

#### 3.2.4. Sorption Isotherms 

**Figure 1:**
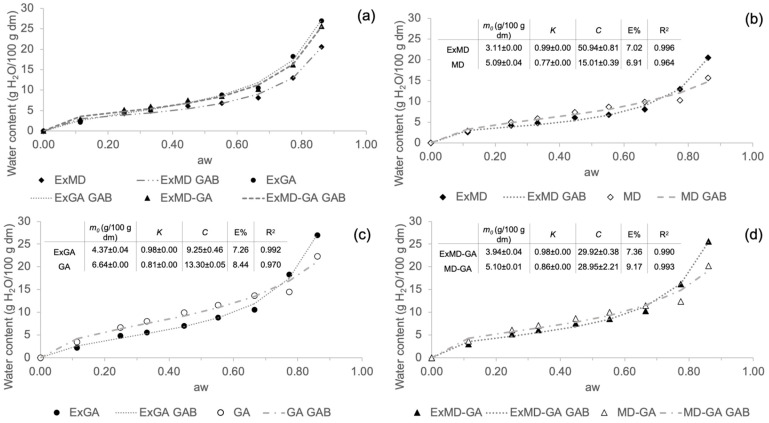
Sorption isotherms at 25 °C of the freeze-dried carriers (reference) and of the corresponding encapsulated hop extracts. (**a**) Comparison of the differently formulated reference freeze-dried powders without the extract (solid symbols); (**b**) microencapsulated hop extract made of maltodextrin and its reference; (**c**) microencapsulated hop extract made of Arabic gum and its reference; (**d**) microencapsulated hop extract made of maltodextrin and Arabic gum mix (ratio 1:1; *w*/*w*) and its reference. Data have been fitted with the GAB model (dashed and dotted lines), and fitting parameters are reported in inserts.

In Figure 1, sorption isotherms of hop microencapsulated extracts and their corresponding encapsulating agents are reported. Water sorption isotherm analysis describes the increase in the moisture content under increasing relative humidity conditions at a constant temperature. All the encapsulated samples show a sigmoidal shape curve (Figure 1a), similar to the so-called “Type 2” most frequently found in food products containing carbohydrates [48]. It is possible to observe that all the encapsulated samples show an increase in water content at increasing water activity. The equilibrium moisture content in powders containing gum Arabic was higher than those made only with maltodextrin, especially at higher water activity conditions (a_w_ > 0.6). Similar behaviour was also observed in rosemary essential oil [44] and in maqui extract [49] microencapsulated using the same coating materials. Comparing the sorption isotherms of the encapsulated hop extracts and the corresponding controls (freeze-dried carrier without extract) (Figure 1b–d), it is possible to observe that the former show lower water adsorption, up to a_w_ ~ 0.7. This result could be attributed to the formation of complexes between the carrier and the extract during the microencapsulation process, which have a higher hydrophobic nature than the sole carrier [14]. These complexes may originate from the establishment of hydrogen bonds and intermolecular weak interactions between, respectively, maltodextrins and Arabic gum constituents with hop phenolic fraction [50,51] and hop’s hydrophobic compounds (e.g., bitter acids) with the Arabic gum hydrophobic protein fraction [52].

Sorption isotherm data were very well fitted by the GAB equation, whose estimated parameters (*m*_0_, *C*, and *K*) are reported in Figure 1 (inserts b, c, and d). The GAB model provides important information through the prediction of the monolayer water content (*m*_0_) important for the stability of dehydrated foods. The parameters from the GAB model fitting should fall within a certain range in order to obtain a satisfactory description of the sigmoidal type isotherm. The *K* values must be 0.24 < *K* ≤ 1 and *C* > 5.67. Keeping *K* and *C* within these ranges assures that the calculated *m*_0_ differs by not more than ±15.5% from the true monolayer water content [53]. In the present study, for all samples, both *K* and *C* parameters fall within this range, which allows a good estimation of the *m*_0_ values_,_ which were <7 g/100 g dm in all different microencapsulated powders. Comparing the *m*_0_ calculated for encapsulated extracts and their control samples, lower values were found in the samples containing the extract.

The *m*_0_ values of the hop encapsulated extracts were similar to those shown for maqui extract encapsulated with 10% of the same coating materials [49] and lower than those reported for beetroot pigment encapsulated by freeze-drying with maltodextrin [54] and for β-carotene encapsulated with Arabic gum and maltodextrins [55]. These differences reveal how the different nature of encapsulated bioactive compounds may affect the moisture content of the monolayer.

#### 3.2.5. Thermal Properties

**Figure 2:**
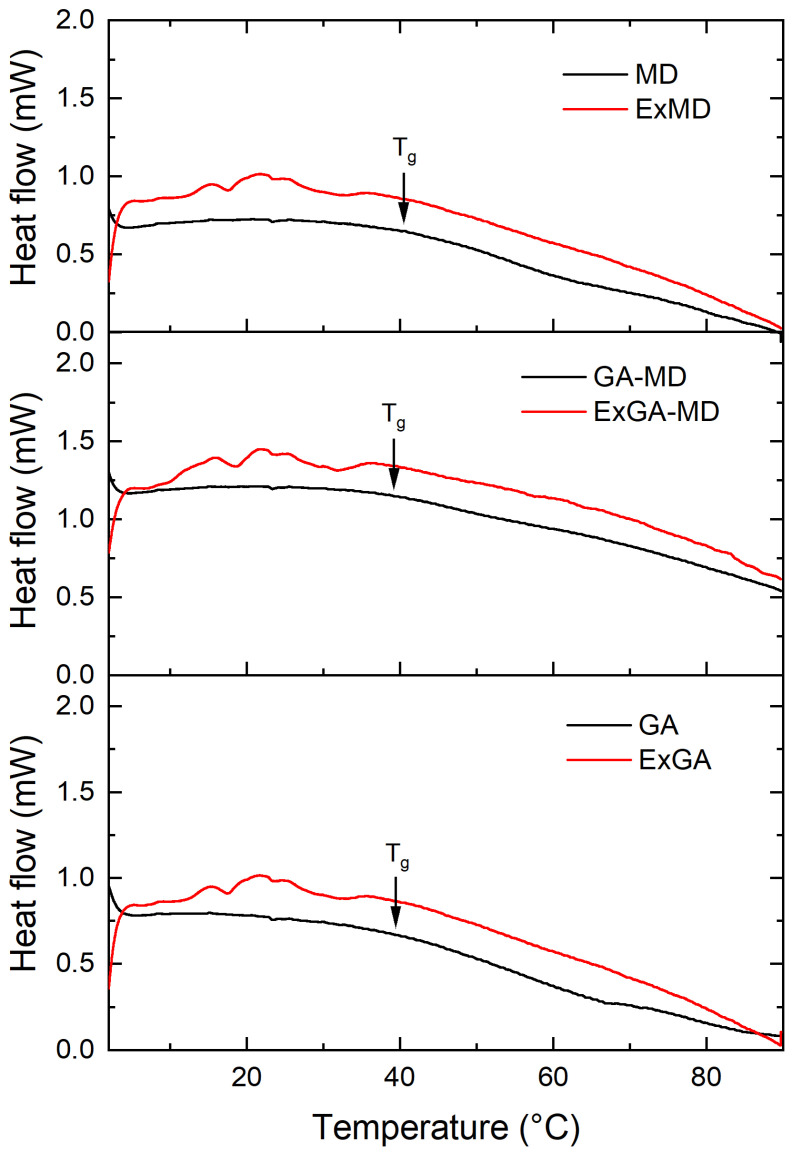
Thermograms of microencapsulated hop extract made of different carriers (red lines) and their references (without extract) (black lines) equilibrated at a_w_ 0.55.

Thermal analysis was carried out on both microencapsulated extracts and corresponding reference samples (without extract) equilibrated at different relative humidity (RH%). All microencapsulated extracts showed an irregular heat flow signal in the proximity of the Tg of the corresponding reference samples (MD, GA, and MD-GA), and this prevented the Tg of most of the microencapsulated hop extract powders from being determined. As an example, in Figure 2 were reported the thermograms of ExMD, ExMD-GA, and ExGA samples and their reference systems equilibrated at RH 55%. 

At the same RH value, microencapsulated extracts for which the glass transition was observed showed Tg values lower or equal to the reference samples (without extract). In particular, at 33% RH, ExGA presented a Tg of 59.7 °C vs. 64.2 °C of GA, and at 44% RH, of 49.5 °C vs. 52.9 °C; conversely, ExMD-GA and MD-GA samples equilibrated at 44% RH and ExMD and MD samples equilibrated at 66% RH showed a similar (*p* > 0.05) Tg (~47 and 30 °C, respectively). Since microencapsulated extracts based on their lower water content (Figure 1) were expected to have a higher Tg than their corresponding reference samples, it can be assumed that the hop extract acted in the powder as a plasticizing agent.

By comparing the carriers under investigation, gum arabic and maltodextrin showed similar Tg values at the different RH they were equilibrated. The Tg values of the MD sample were in accordance with data reported in the literature for maltodextrin of similar DE [56]. Concerning the MD-GA sample, a lower Tg value was found with respect to both the single components at all the RH tested. With respect to MD, this result was apparently related exclusively to the higher moisture content (Figure 1b,d). Conversely, with respect to the GA sample, MD-GA showed a lower Tg value despite the higher moisture content (Figure 1b,c). 

To gain further insight into the thermal and chemico-physical properties of the carriers used to produce the encapsulated carriers and better understand these results, the experimental Tg data of the carriers were fitted by the Gordon–Taylor equation. GA showed the highest Tg_dry_ and also the highest k value, indicating a higher plasticizing effect of water on the matrix, i.e., stronger interaction between water and this solid, see estimated parameters for each carrier reported in the Supplementary Material (Table S1). By comparing the Gordon–Taylor parameters, it can be also noted that the maltodextrin affected much more than the Arabic gum the plasticization effect of water on MD-GA and that MD-GA showed a Tg_dry_ lower than its single constituents. This unexpected result supports the experimental data and could be related to the partial immiscibility exhibited by the high molecular weight components (i.e carbohydrates, proteins) composing the complex MD-GA systems with consequent formation of phases featuring different physicochemical properties [57].

#### 3.2.6. Morphology 

**Figure 3:**
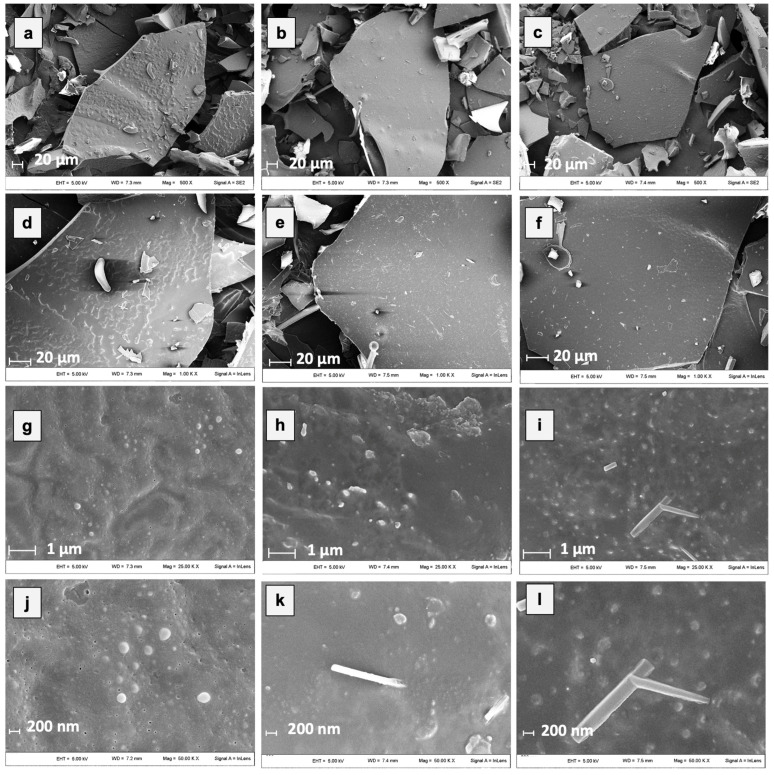
Micrographs of different microencapsulated hop extracts at different magnifications. In detail: ExMD (**a**,**d**,**g**,**j**); ExGA (**b**,**e**,**h**,**k**), and ExMD-GA (**c**,**f**,**i**,**l**).

Microstructural properties of freeze-dried powders are primarily formed during freezing and affect the porosity and strength of solids of freeze-dried foods as well as the entrapment of functional food components [58]. In Figure 3, SEM micrographs of hop extracts encapsulated by freeze-drying with 100% MD, GA, and MD in combination with GA (1:1) are shown. 

All the samples after freeze-drying showed a porous and glassy matrix containing air cells whose size and shape depend on the processing conditions used and the composition of the initial system. By grinding, powders with sheet-like particles of irregular size and shape were produced, preserving partly the initial cell-like structure and wall characteristics. However, by observing the micrographs at the highest magnification (Figure 3j–l), some microstructural differences among the powders could be observed. In particular, the ExGA particles showed a more continuous and compact structure with smoother surfaces compared to the ones of ExMD, which, on the contrary, presented a rougher and more porous surface morphology. Moreover, despite all the samples showing on their surface spherical spots ascribable to the presence of the hop extract [21], the differences observed in relation to their amount, size, and distribution arise from the carrier’s chemical properties, which lead to a different dissolution of the hop extract in the matrix structure.

### 3.3. Load Yield, Encapsulation Efficiency, Bitter Acid Content, and Antioxidant Capacity of Encapsulated Hop Extracts

The total phenolic content (surface + encapsulated) of the hop extract microencapsulated using different wall materials is shown in Table 2. Compared to the concentrated hop extracts (Table 1), all microencapsulated extracts showed a lower amount of antioxidant compounds due to the dilution effect given by the carrier addition. The TPC of the encapsulated extracts varied from 10.3 to 13.2 mg GAE g^−1^ with the ExMD and ExGA samples showing the lowest and highest values, respectively. These results are in agreement with those obtained on a polyherbal formulation [59] and on a model fruit juice microencapsulated by freeze-drying with Arabic gum and maltodextrins (DE 12 and 20) [60].

The load yield (Y) (i.e., the TPC % retained in the powders after freeze-drying) of the three samples ranges from 58 to 67% with ExGA and ExMD-GA samples presenting the highest values. The Y value of ExMD was lower than that measured for olive leaf extracts encapsulated by freeze-drying with maltodextrins of equal DE [21], for durian flavour [61], and for cloudberry (*Rubus chamaemorus*) phenolics encapsulated by freeze-drying with maltodextrins of different DE values [14]. The TPC losses observed in the freeze-dried samples may be attributed to the chemical degradation (hydrolysis, oxidation) of phenolic compounds during the preparation of hop extract water dispersions and during freeze-drying and sample powdering [14,21] as well as to the precipitation during the solubilization, centrifugation, and filtration of the encapsulated hop extracts of poorly water-soluble compounds with reducing activity capable to react with the Folin–Ciocalteu reagent. Indeed, the higher TPC and Y value of the samples containing Arabic gum could be explained by the lower losses of these compounds during the above-mentioned operations carried out after freeze-drying due to their higher solubilization and dispersion promoted by the emulsifying properties of Arabic gum and its effect on water polarity [52,62]. Moreover, the surface porosity observed by microstructure analysis (Figure 3) in the ExMD powder could have favoured the oxidation of phenolic compounds present on the surface of the particles.

As regards the encapsulation efficiency (Table 2), defined as the percentage of phenolic compounds (TPC) entrapped in the matrix of wall material after freeze-drying with respect to that present in the whole system (entrapped + surface), the highest value (~79%) was achieved when the sole maltodextrin was used as encapsulating material. Similar results were obtained by other authors [19,47,56,63]. However, Chranioti and Tzia [64] encapsulating by freeze-drying a fennel oleoresin product with Arabic gum and a mix of Arabic gum and maltodextrin (DE 21) obtained the highest encapsulation efficiency when gum arabic was used as single coating material. This different behaviour may be explained by the fact that the encapsulation efficiency is highly dependent on the encapsulated core compounds, the coating material used, and their interactions [65].

Regarding the bitter acids (Table 2), ExGA and ExMD-GA showed a higher (~+34%) α-acids content than ExMD, while the highest content of β-acids was found in the ExGA sample. As for polyphenols, these results could be due to the higher retention of these apolar compounds (acylphloroglucinols) during the sample preparation thanks to their interaction with the hydrophobic proteins fraction of the Arabic gum, which acts as emulsifiers [52].

As concerns the antioxidant capacity (Table 2) measured by both FRAP and ABTS assays, according to the TPC and bitter acids data, the highest and lowest value was registered, respectively, in ExGA and ExMD samples. The higher retention of antioxidant compounds in samples encapsulated by Arabic gum than in maltodextrin was also observed by Hussain et al. [59] and can be attributed to the composition and structure of Arabic gum, which is a highly branched heteropolymer of sugars containing a small amount of protein covalently linked to the carbohydrate chain, acting as an excellent film-forming and emulsifying agent capable to entrap and stabilize bioactive compounds [66].

### 3.4. Storage Stability of Microencapsulated Powders

The storage stability of microencapsulated hop extracts was evaluated at different temperatures 5, 20, 35, and 50 °C. Based on the a_w_ of the samples, the corresponding water content and the experimental Tg values in this study (see Supplementary Material, Table S1) or Tg data reported in the literature for the same carriers equilibrated at similar a_w_ values [56], it can be assumed that all the powders at all the storage temperature were and remained in a glassy state. This physical condition is generally retained to protect the encapsulated component from various deteriorative changes such as oxidation [14].

**Figure 4:**
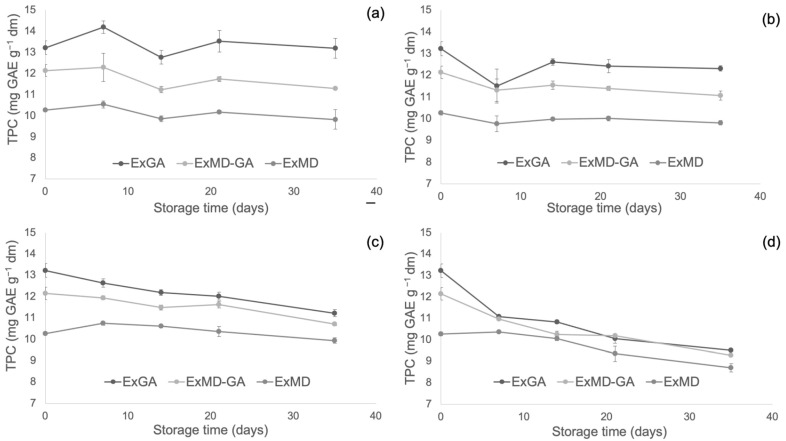
Total phenolic content (TPC) of encapsulated hop extract during storage at different temperatures: 5 °C (**a**), 20 °C (**b**), 35 °C (**c**), 50 °C (**d**).

**Figure 5:**
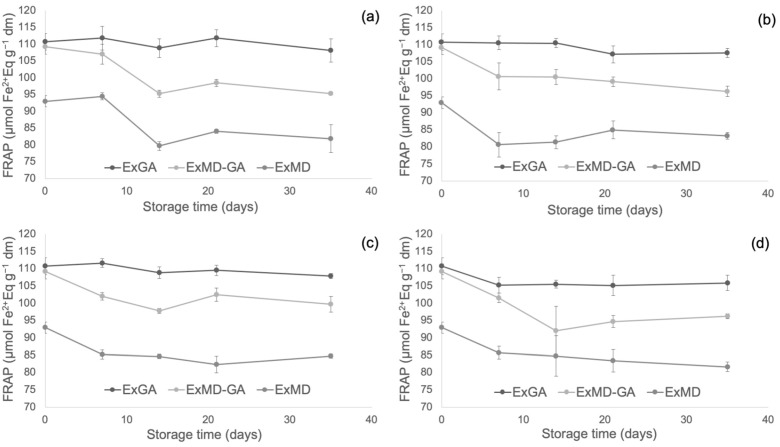
Ferric reducing antioxidant power (FRAP) of encapsulated hop extract during storage at different temperatures: 5 °C (**a**), 20 °C (**b**), 35 °C (**c**), 50 °C (**d**).

**Figure 6:**
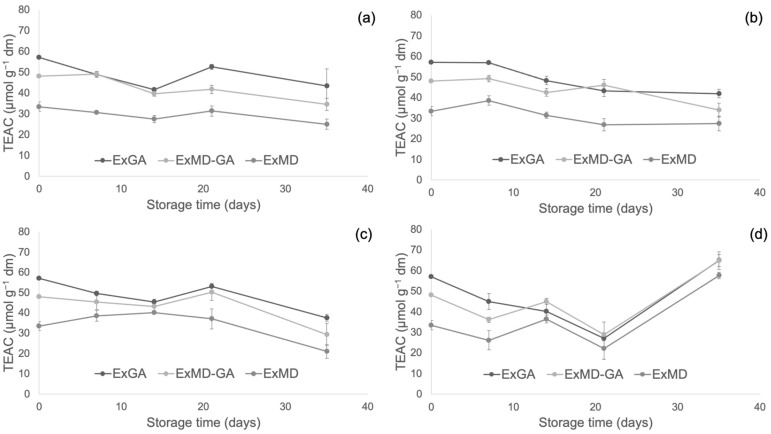
Trolox equivalent antioxidant capacity (TEAC) of encapsulated hop extract during storage at different temperatures: 5 °C (**a**), 20 °C (**b**), 35 °C (**c**), 50 °C (**d**).

Figure 4, Figure 5 and Figure 6 report, respectively, the total phenolic content (TPC) and the antioxidant activity as measured by the FRAP and ABTS assays of the encapsulated hop extracts during storage at different temperatures (for the full dataset, data expressed as loss % and results of the statistical analysis, see Table S2 in Supplementary Material).

Overall, none of the investigated samples showed any significant TPC variation during storage at 5 °C, while at 20 °C a slight loss (10%) was observed after 35 days and only for the ExMD-GA sample. When encapsulated powders were stored at 35 °C, a decrease in the TPC was observed in the ExMD-GA and ExGA samples with a loss, after 35 days, of 12% and 15%, respectively; on the contrary, in the sample encapsulated with maltodextrin (ExMD) no variation (*p* > 0.05) was observed. The TPC storage stability showed by the ExMD sample was similar to that reported for plant extracts encapsulated by freeze-drying and spray-drying with maltodextrins and stored at similar temperatures [37,67].

At the highest storage temperature (50 °C), all samples showed a TPC decrease over time with ExMD presenting after 35 days the lowest loss (15%). These results could be ascribed to the higher fraction of encapsulated phenolic compounds in the ExMD sample compared to those containing Arabic gum and consequent higher protection towards oxidation reactions during storage and exposition at high temperatures.

Concerning the powders’ antioxidant properties, it is possible to observe that irrespective of the storage temperature, the ExGA sample did not highlight any significant (*p* < 0.05) change of the ferric reducing power during storage, while in ExMD and ExMD-GA samples, it decreased to a different extent (9–12%) depending on the storage temperature. 

Conversely, with respect to the ABTS radical scavenging activity, all the powders showed during storage at 5, 20, and 35 °C decreasing values. In particular, after 35 days of storage at 5 and 20 °C, a TEAC reduction in the range of 18–29% was observed with no significant differences (*p* < 0.05) between the samples, while at 35 °C the samples showed, on average, a 37% TEAC loss. However, when samples were stored at 50 °C, fluctuating values were obtained over time. This trend was also observed in white wine [68], and it could be due to the oxidation of phenolic compounds such as the flavanols catechin and epicatechin and polymerization reactions with the formation of new compounds with higher antiradical activity [69]. The discrepancies between the results of the FRAP and ABTS assays can be related to the different mechanisms of action of the two assays towards the antioxidants present in the extracts, including isoxanthohumol, which has been reported to have a higher response to the ABTS assay than to the FRAP assay [70]. Overall, by observing data collected by TPC, ABTS, and FRAP assays, it can be highlighted that the sample encapsulated with maltodextrin (ExMD), despite the highest percentage of encapsulated phenols, presented after storage a lower (*p* < 0.05) phenolic content and antioxidant activity compared to the powders encapsulated with Arabic gum. This result could be related to the high surface porosity of the ExMD powder (Figure 3) that could have favoured the oxidation of phenolic compounds present on the surface of the particles during storage.

In Table 3, the content of bitter acids in microencapsulated hop extracts before and after 35 days of storage at different temperatures is shown. Overall, all the samples showed a significant reduction (*p* < 0.05) in both α- and β-acids, especially for storage temperatures higher than 35 °C (*p* < 0.05). Among the bitter acids, the β-acids showed a higher sensitivity to temperature than α-acids in agreement with Krofta et al. [71]. By comparing the bitter acid content among the differently microencapsulated samples, no significant differences could be observed with respect to α-acids for samples stored at 20 °C and 35 °C, while for those stored at 5 °C and 50 °C, the ExGA sample showed overall a higher content and a lower loss percentage than the ExMD sample (21% vs. 27% at 5 °C and 78% vs. 81% at 50 °C). Conversely, with respect to β-acids, the ExMD sample, despite the lower initial content, was the sole sample still retaining (13%) these compounds after 35 days of storage at 50 °C. These results indicate a different interaction of the investigated carriers with α- and β-acids and possibly a different entrapment capability. The results obtained for β-acids agree, in particular, with those related to the phenolic fraction and suggest a higher capability of maltodextrin to encapsulate and protect sensitive compounds from the exposition to oxidating agents such as oxygen.

### 3.5. Effect of Microencapsulation on the Retention of Hop Antioxidant Compounds and Properties

**Figure 7:**
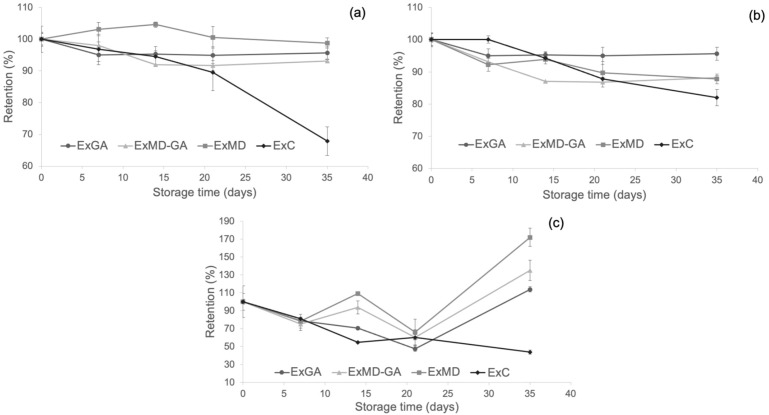
Changes of (**a**) TPC; (**b**) FRAP antioxidant activity; (**c**) ABTS antioxidant activity of the microencapsulates and the concentrated hop extract at 50 °C during storage. Data are expressed in retention percentage with respect to the initial value of the non-stored samples.

With the aim to evaluate the protective effect of microencapsulation on the phenolic compounds, bitter acids, and antioxidant properties of the hop extract, the retention of these parameters in concentrated hop extract (ExC) during storage at 50 °C was also determined and compared with that of microencapsulated extracts. This temperature was chosen since the degradation of antioxidant compounds in encapsulated hop extracts occurred mostly during storage at this temperature. The resulting data are reported in Figure 7.

As shown (Figure 7a), microencapsulation with maltodextrin allowed the preservation of the phenolic fraction of the hop extract during storage, but with respect to the other carriers, this effect was observed only at the longest storage time. The protective effect of microencapsulation with MD and AG towards phenolic compounds was found also by other authors [38,72], and it is ascribed to the wall effect exerted by these encapsulation materials [73].

As concerns the antioxidant activity (AOA) as determined by the FRAP (Figure 7b) and ABTS assays (Figure 7c), a positive effect of freeze-drying microencapsulation was observed mostly at the longest storage time with encapsulated samples showing higher retention values compared to ExC irrespective of their formulation.

Finally, by evaluating the content of α-and β- acids of the ExC sample after 35 days at 50 °C and comparing it with that of the microencapsulated powders (Table 3), it was observed as the former with 16.45% (*w*/*w*) of α-acids and 2.83% (*w*/*w*) of α-acids highlighted a lower loss (61% on average) of bitter acids compared to the microencapsulated extracts. These results could be due to the high density and viscosity of the resinous extract, which may have hindered the oxygen diffusion into the matrix and the occurrence of oxidative reactions. 

## 4. Conclusions

In this study, freeze-drying microencapsulation of hop extract by using different coating materials (single component vs. binary maltodextrin-Arabic gum mix) was tested with the aim to develop a new ingredient with different techno-functional properties and stability for the production of high-quality, clean-label, and functional food products. 

The type of coating material significantly affected the physical properties of the powders, affecting colour, bulk density, thermal properties, and their interaction with water. Retention of phenolic compounds and bitter acids was affected as well by the carrier material during both processing and storage. In particular, the use of Arabic gum as the wall material provided the highest yield of phenolic compounds and bitter acids after processing, while maltodextrin allowed a higher encapsulation and retention of these compounds during storage at high temperatures. These differences arose mainly from the different chemical properties of the carriers interacting to different extents with the core material leading to more or less homogeneous matrices.

Further studies are required to investigate the technological applications of microencapsulated hop extracts in complex food formulations and to evaluate their impact on quality and sensory attributes (e.g., bitterness), and microbiological and chemico-physical stability during storage.

## Figures and Tables

**Table 1:** Characterization of concentrated hop extract.

	TPC	FRAP	TEAC	α-Acids	β-Acids
	(mg GAE g ^−1^ dm)	(µmol Fe^2+^Eq g ^−1^ dm)	(µmol g^−1^ dm)	(% *w*/*w*)	(% *w*/*w*)
ExC	158 ± 1 (83.5 ± 0.7)	1284 ± 53 (528 ± 28)	757 ± 20 (401 ± 11)	40.7 ± 0.5	7.53 ± 0.11

ExC: Concentrated hop extract; TPC: Total phenolic content (mg GAE g^−1^ dm); FRAP: Ferric reducing antioxidant power (µmol Fe^2+^Eq g ^−1^ dm); TEAC: Trolox equivalent antioxidant capacity (µmol g^−1^ dm); dm: dry matter. Data in parenthesis relate to dried hop cones.

**Table 2:** Physico-chemical, colouring, antioxidant properties and composition of the differently microencapsulated hop extracts.

Parameters	ExGA	ExMD-GA	ExMD
Water activity (a_w_)	0.095 ^b^ ± 0.003	0.185 ^a^ ± 0.007	0.085 ^b^ ± 0.005
Moisture content (%)	4.07 ^a^ ± 0.64	5.27 ^a^ ± 0.05	2.19 ^b^ ± 0.28
Solubility (%)	99.10 ^a^ ± 0.01	99.20 ^a^ ± 0.2	99.10 ^a^ ± 0.01
Bulk density (g/mL)	0.236 ^a^ ± 0.011	0.159 ^c^ ± 0.002	0.172 ^b^ ± 0.005
Tapped density (g/mL)	0.395 ^a^ ± 0.004	0.269 ^c^ ± 0.015	0.322 ^b^ ± 0.009
Carr Index (CI)	38.5 ^a^ ± 2.2	41.5 ^a^ ± 2.3	45.5 ^a^ ± 3.1
Hausner Ratio (HR)	1.63 ^a^ ± 0.06	1.71 ^a^ ± 0.07	1.84 ^a^ ± 0.10
Lightness (L*) ^1^	67.3 ^c^ ± 0.2	70.7 ^b^ ± 0.6	80.5 ^a^ ± 0.4
(a*) ^1^	−2.18 ^a^ ± 0.17	−2.66 ^b^ ± 0.15	−4.75 ^c^ ± 0.25
(b*) ^1^	31.4 ^c^ ± 0.5	35.1 ^b^ ± 0.3	37.8 ^a^ ± 0.6
Hue angle (h°) ^1^	94.5 ^b^ ± 1.1	94.4 ^b^ ± 0.2	97.1 ^a^ ± 0.4
Lightness (L*) ^2^	95.7 ^c^ ± 0.0	96.0 ^b^ ± 0.1	96.3 ^a^ ± 0.0
(a*) ^2^	−1.48 ^b^ ± 0.02	−1.28 ^a^ ± 0.10	−1.44 ^b^ ± 0.02
(b*) ^2^	5.34 ^a^ ± 0.11	4.45 ^b^ ± 0.42	4.49 ^b^ ± 0.08
Hue angle (h°) ^2^	105.2 ^c^ ± 0.1	106.3 ^b^ ± 0.0	107.7 ^a^ ± 0.0
TPC (mg GAE g^−1^ dm)	13.23 ^a^ ± 0.32	12.15 ^b^ ± 0.29	10.27 ^c^ ± 0.09
FRAP (µmol Fe^2+^ g^−1^ dm)	110.8 ^a^ ± 2.4	109.2 ^a^ ± 2.1	93.0 ^b^ ± 1.7
TEAC (µmol g^−1^ dm)	57.83 ^a^ ± 3.78	48.57 ^b^ ± 2.66	36.63 ^c^ ± 1.90
EE (%)	73.5 ^b^ ± 1.1	64.3 ^c^ ± 1.4	78.5 ^a^ ± 2.2
Y (%)	66.6 ^a^ ± 3.1	62.5 ^a^ ± 3.1	58.1 ^b^ ± 2.4
α-acids (%*w*/*w*)	2.78 ^a^ ± 0.22	2.74 ^a^ ± 0.04	2.06 ^b^ ± 0.05
β-acids (%*w*/*w*)	0.53 ^a^ ± 0.07	0.28 ^b^ ± 0.04	0.15 ^c^ ± 0.02

^1^ colour; ^2^ colouring power; TPC: Total Phenolic Content (mg GAE g^−1^ dm); FRAP: Ferric reducing antioxidant power (µmol Fe^2+^Eq g ^−1^ dm); TEAC: Trolox equivalent antioxidant capacity (µmol g^−1^ dm); EE: encapsulation efficiency; Y: load yield; ExGA: microencapsulated hop extract with Arabic gum; ExMD-GA: microencapsulated hop extract with mixture of maltodextrin and Arabic gum; ExMD: microencapsulated hop extract with maltodextrin; dm: dry matter. Data on rows with different letters are statistically different at *p* level < 0.05.

**Table 3:** Alpha and Beta acids content of differently microencapsulated hop extracts before and after 35 days of storage at different temperatures.

Storage		α-Acids (%*w*/*w*)			β-Acids (%*w*/*w*)		
Time (days)	Temperature (°C)	ExGA	ExMD-GA	ExMD	ExGA	ExMD-GA	ExMD
0	-	2.78 ^aA^ ± 0.22 (-)	2.74 ^aA^ ± 0.04 (-)	2.06 ^bA^ ± 0.05 (-)	0.53 ^aA^ ± 0.07 (-)	0.28 ^bA^ ± 0.04 (-)	0.15 ^cA^ ± 0.02 (-)
35	5	2.20 ^aAB^ ± 0.25 (20.69 ± 9.08)	1.73 ^abB^ ± 0.28 (40.05 ± 12.04)	1.49 ^bB^ ± 0.05 (27.48 ± 2.60)	0.20 ^aBC^ ± 0.08 (62.66 ± 14.78)	0.11 ^aB^ ± 0.06 (70.45 ± 12.37)	0.04 ^aBC^ ± 0.03 (57.48 ± 0.59)
35	20	1.69 ^aB^ ± 0.55 (50.26 ± 6.87)	1.62 ^aB^ ± 0.33 (45.45 ± 12.68)	1.47 ^aB^ ± 0.05 (28.50 ± 2.49)	0.11 ^aCD^ ± 0.10 (90.75 ± 4.44)	0.13 ^aB^ ± 0.09 (64.48 ± 36.99)	0.10 ^aAB^ ± 0.04 (19.10 ± 9.39)
35	35	1.47 ^aBC^ ± 0.49 (37.54 ± 8.83)	1.03 ^aC^ ± 0.01 (62.07 ± 0.49)	1.33 ^aC^ ± 0.03 (35.18 ± 1.50)	0.30 ^aB^ ± 0.01 (43.94 ± 1.12)	0.05 ^cB^ ± 0.00 (82.54 ± 4.98)	0.11 ^bAB^ ± 0.00 (29.50 ± 0.90)
35	50	0.60 ^aC^ ± 0.03 (78.42 ± 1.24)	0.59 ^aC^ ± 0.05 (77.92 ± 0.62)	0.38 ^bD^ ± 0.02 (81.39 ± 1.07)	0.00 ^bD^ ± 0.00 (100.00 ± 0.00)	0.00 ^bB^ ± 0.00 (100.00 ± 0.00)	0.02 ^aC^ ± 0.00 (87.31 ± 3.28)

ExGA: microencapsulated hop extract with Arabic gum; ExMD-GA: microencapsulated hop extract with mixture of maltodextrin and Arabic gum; ExMD: microencapsulated hop extract with maltodextrin. Data reported in parenthesis are loss percentages with respect to the initial content. Data in rows with different lowercase letters are statistically different at *p* level < 0.05. Data in columns with different capital letters are statistically different at *p* level < 0.05.

## Data Availability

Data is contained within the article and Supplementary Material.

## Supplementary Materials

The following supporting information can be downloaded at https://www.mdpi.com/article/10.3390/antiox12020442/s1, Figure S1: Procedure for the preparation of the encapsulated hop extracts; Table S1: Estimated parameters of Gordon–Taylor model left block and Tg measured at different aw for reference powders made of maltodextrin, maltodextrin-gum arabic, and gum arabic. Table S2: Phenolic content and antioxidant activity of differently microencapsulated hop extract during storage at different temperatures.
