# Supplementary material for: Freeze-Drying Microencapsulation of Hop Extract: Effect of Carrier Composition on Physical, Techno-Functional, and Stability Properties
Source: Antioxidants (Basel). 2023 Feb 10;12(2):442. doi: 10.3390/antiox12020442 (PMC9951912; doi:10.3390/antiox12020442)
Supplement: Supplementary file 1 [file antioxidants-12-00442-s001.zip › Table S1.pdf]

Supplementary Table S1. *Estimated parameters of Gordon-Taylor model left block and Tg measured at different a_w for reference powders made of maltodextrin, maltodextrin-gum arabic and gum arabic;*

	**Tg (°C)**	***k***	**R^2**	**E (%)**	**Tg(°C)**		
					a_w0.44	a_w0.53	a_w0.65
**MD**	131.86	6.15	0.994	6.74	53.04	37.71	31.03
**MD-GA**	99.48	3.70	0.999	3.40	47.17	38.03	28.14
**GA**	164.55	6.62	0.999	2.34	52.93	37.94	23.02
