# Supplementary material for: Freeze-Drying Microencapsulation of Hop Extract: Effect of Carrier Composition on Physical, Techno-Functional, and Stability Properties
Source: Antioxidants (Basel). 2023 Feb 10;12(2):442. doi: 10.3390/antiox12020442 (PMC9951912; doi:10.3390/antiox12020442)
Supplement: Supplementary file 1 [file antioxidants-12-00442-s001.zip › Table S2.pdf]

Table S2: Phenolic content and antioxidant activity of differently microencapsulated hop extract during storage at different temperatures.

		TPC (mg GAE g^{-1} dm)			FRAP (µmol Fe^{2+} g^{-1} dm)			TEAC (µmol g^{-1} dm)		
storage time (days)	storage temperature (°C)	ExGA	ExMD-GA	ExMD	ExGA	ExMD-GA	ExMD	ExGA	ExMD-GA	ExMD
0	-	13.23^{abA}± 0.32<br>(-)	12.15^{abB}± 0.29<br>(-)	10.27^{abC}± 0.09<br>(-)	110.75^{aA}±2.46<br>(-)	109.17^{aA}± 2.14<br>(-)	92.96^{aB}± 1.66<br>(-)	57.83^{aA}±0.55<br>(-)	48.57^{aB}±0.41<br>(-)	36.63^{aC}±2.26<br>(-)
7	5	14.20^{aA}± 0.30<br>(+7.34±2.27)	12.30^{aB}± 0.66<br>(+3.98±3.71)	10.54^{aC}± 0.17<br>(+2.65±1.61)	111.76^{aA}± 3.46<br>(+0.92±3.13)	107.02^{aB}± 2.95<br>(1.97±2.71)	94.47^{aC}± 1.09<br>(+1.62±1.17)	48.89^{bcA}±1.48<br>(14.29±2.59)	48.99^{aA}±0.13<br>(+2.07±0.27)	30.53^{bB}±0.03<br>(8.68±0.09)
14	5	12.77^{bA} ± 0.32<br>(2.05±0.10)	11.24^{cB}± 0.15<br>(7.45±1.27)	9.86^{bC}± 0.14<br>(4.04±1.36)	108.84^{aA}± 2.79<br>(1.73±2.52)	95.24^{bB}± 1.02<br>(12.76±0.93)	79.69^{bC}± 1.29<br>(14.28±1.39)	41.61^{cA}±0.61<br>(21.99±8.79)	41.41^{bA}±1.12<br>(13.73±6.96)	27.52^{bcB}±1.75<br>(17.67±5.24)
21	5	13.53^{abA}± 0.51<br>(+2.26±3.86)	11.75^{abcB} ±0.12<br>(3.26±0.98)	10.18 ^{abC}± 0.08<br>(0.92±0.77)	111.79^{aA}± 2.55<br>(+0.93±2.30)	98.47^{bB}± 1.04<br>(9.81±0.95)	84.07^{bC}± 0.65<br>(9.57±0.70)	52.52^{abA}±1.31<br>(7.92±2.30)	41.77^{bB}±2.04<br>(12.97±4.26)	31.29^{bC}±2.45<br>(6.40±4.26)
35	5	13.20^{abA}± 0.47<br>(0.21±3.55)	11.29^{bcB}± 0.01<br>(7.06±0.11)	9.83^{bC}± 0.47<br>(1.78±1.72)	108.08^{aA}± 3.46<br>(2.41±3.12)	95.34^{bB}± 0.44<br>(12.67±0.40)	81.88^{bC}± 4.11<br>(9.44±1.43)	43.33^{cA}±8.24<br>(24.04±14.44)	34.50^{cB}±2.9<br>(28.13±6.15)	24.96^{cC}±2.40<br>(25.34±7.18)
0	-	13.23^{aA} ± 0.32<br>(-)	12.15^{aB}± 0.29<br>(-)	10.27^{aC} ± 0.09<br>(-)	110.75^{aA}±2.46<br>(-)	109.17^{aA}± 2.14<br>(-)	92.96^{aB}± 1.66<br>(-)	57.83^{aA}±0.55<br>(-)	48.57^{abB}±0.41<br>(-)	36.63^{abC}±2.26<br>(-)
7	20	11.52^{bA}± 0.78<br>(16.34±0.23)	11.33^{bA}± 0.51<br>(4.48±1.98)	9.78^{bB}± 0.36<br>(4.89±3.50)	110.52^{aA}± 2.02<br>(0.21±1.82)	100.68^{bB}± 3.95<br>(5.85±2.00)	80.65^{bC}± 3.58<br>(11.03±0.31)	57.02^{aA}±0.22<br>(0.05±0.38)	49.18^{aB}±1.64<br>(+2.46±0.38)	38.56^{aC}±2.31<br>(+15.33±6.90)
14	20	12.62^{abA}± 0.16<br>(4.62±1.19)	11.55^{abB}±0.19<br>(4.91±1.57)	9.98^{abC}± 0.04<br>(2.84±0.40)	110.49^{aA}± 1.37<br>(0.23±1.24)	100.50^{bB}± 2.22<br>(7.95±2.03)	81.36^{bC}± 1.88<br>(12.48±2.02)	47.34^{bA}±2.05<br>(15.38±3.60)	42.52^{bB}±1.84<br>(11.41±3.83)	31.4^{bcC}±1.49<br>(5.99±4.44)
21	20	12.44^{abA}± 0.30<br>(6.06±2.27)	11.41^{abB}± 0.10<br>(6.09±0.85)	10.02^{abC}± 0.11<br>(2.48±1.02)	107.16^{aA}± 2.53<br>(3.25±2.29)	99.13^{bB}±1.42<br>(9.20±1.30)	84.94^{bC}± 2.62<br>(8.62±2.82)	43.27^{bA}±2.87<br>(24.15±5.04)	46.09^{abA}±2.65<br>(3.98±5.52)	26.78^{cB}±2.94<br>(19.90±8.79)
35	20	12.32^{abA}± 0.12<br>(6.84±0.88)	11.07^{bB}±0.21<br>(8.89±1.75)	9.82^{abC} ± 0.11<br>(4.37±1.04)	107.53^{aA}± 1.32<br>(2.90±1.19)	96.31^{bB}± 1.48<br>(11.79±1.36)	83.22^{bC}± 0.97<br>(10.48±1.04)	41.94^{bA}±2.01<br>(26.48±3.55)	33.94^{cB}±3.29<br>(29.28±6.86)	27.38^{cC}±3.58<br>(18.10±10.72)
0	-	13.23^{aA} ± 0.32<br>(-)	12.15^{aB}± 0.29<br>(-)	10.27^{bcC} ± 0.09<br>(-)	110.75^{aA}±2.46<br>(-)	109.17^{aA}± 2.14<br>(-)	92.96^{aB}± 1.66<br>(-)	57.83^{aA}±0.55<br>(-)	48.57^{aB}±0.41<br>(-)	36.63^{aC}±2.26<br>(-)
7	35	12.65^{bA}± 0.19<br>(4.41±1.47)	11.95^{abB}± 0.08<br>(1.66±2.20)	10.75^{aC}± 0.09<br>(+4.67±0.87)	111.63^{aA}± 1.28<br>(+0.80±1.16)	102.04^{bcB}± 1.10<br>(6.53±1.00)	85.22^{bC}±1.31<br>(8.33±1.41)	49.52^{bcA}±1.09<br>(13.20±1.92)	45.34^{aAB}±3.54<br>(5.53±7.38)	35.01^{aB}±2.57<br>(+15.06±7.70)
14	35	12.18^{bcA}± 0.14<br>(7.90±1.03)	11.49^{cB}± 0.13<br>(5.41±1.03)	10.61^{abC}± 0.08<br>(+3.34±0.75)	108.84^{aA}± 1.66<br>(1.73±1.50)	97.80^{cB}± 0.75<br>(10.42±0.69)	84.67^{bC}± 0.63<br>(8.92±0.68)	45.30^{cA}±0.01<br>(20.58±0.03)	43.14^{aAB}±3.54<br>(10.12±7.34)	40.06^{aB}±0.23<br>(+19.84±0.69)
21	35	12.02^{cA}± 0.19<br>(9.13±1.42)	11.63^{bcA}± 0.15<br>(4.23±1.26)	10.37^{bB}± 0.24<br>(+0.92±2.33)	109.54^{aA}± 1.48<br>(1.09±1.34)	102.49^{bB}± 1.90<br>(6.12±1.74)	82.36^{bC}± 2.40<br>(11.41±2.58)	52.96^{abA}±1.72<br>(7.15±3.01)	50.18^{aA}±3.99<br>(+4.55±8.32)	37.02^{aB}±4.96<br>(+10.75±14.8)
35	35	11.23^{dA}± 0.17<br>(15.15±1.28)	10.71^{dB}± 0.07<br>(11.88±0.61)	9.94^{cC}± 0.13<br>(3.22±1.27)	107.89^{aA}± 0.77<br>(2.59±0.70)	99.70^{bcB}± 2.25<br>(8.68±2.06)	84.73^{bC}± 0.60<br>(8.86±0.64)	37.57^{dA}±1.71<br>(34.14±2.99)	29.32^{bB}±5.49<br>(38.92±11.44)	20.95^{bC}±3.36<br>(37.33±10.05)
0	-	13.23^{aA} ± 0.32<br>(-)	12.15^{aB}± 0.29<br>(-)	10.27^{aC} ± 0.09<br>(-)	110.75^{aA}±2.46<br>(-)	109.17^{aA}± 2.14<br>(-)	92.96^{aB}± 1.66<br>(-)	57.83^{aA}±0.55<br>(-)	48.57^{bB}±0.41<br>(-)	36.63^{bC}±2.26<br>(-)
7	50	11.09^{bA} ± 0.06<br>(16.19±0.44)	10.97^{bA}± 0.07<br>(9.68±0.59)	10.37^{aB}± 0.05<br>(+0.99±0.48)	105.25^{aA}± 2.32<br>(4.97±2.10)	101.58^{abA}± 1.35<br>(6.96±1.24)	85.72^{abcB}± 1.87<br>(7.79±2.01)	44.96^{bA}±3.81<br>(21.18±6.68)	36.07^{cdAB}±1.30<br>(24.86±2.71)	29.03^{bB}±4.71<br>(21.97±14.10)
14	50	10.83^{bA}± 0.09<br>(18.12±0.65)	10.24^{cB}± 0.17<br>(15.71±1.39)	10.05^{aB}± 0.09<br>(2.13±0.89)	105.53^{aA}± 1.10<br>(4.71±0.99)	90.87^{cB}± 7.09<br>(13.02±0.09)	90.60^{abB}± 5.79<br>(6.13±0.70)	43.35^{bA}±6.26<br>(29.65±7.02)	44.97^{bcA}±1.46<br>(6.32±3.04)	34.05^{bA}±4.31<br>(+8.96±5.42)
21	50	10.05^{cA}± 0.21<br>(24.00±1.57)	10.20^{cA}± 0.05<br>(16.07±0.38)	9.35^{bB}± 0.36<br>(8.98±3.49)	105.18^{aA}± 2.91<br>(5.04±2.63)	94.75^{bcB}±1.68<br>(13.21±1.54)	83.43^{bcC}± 3.19<br>(10.26±3.44)	29.47^{cA}±1.37<br>(52.46±2.41)	32.92^{dA}±6.18<br>(39.85±12.87)	28.24^{bA}±5.07<br>(34.19±15.15)
35	50	9.51^{dA}± 0.05<br>(28.12±0.37)	9.28^{dA}± 0.02<br>(23.62±0.14)	8.70^{cB}± 0.19<br>(15.31±1.90)	105.90^{aA}± 2.22<br>(4.38±2.01)	96.22^{bcB}±0.74<br>(11.87±0.67)	81.66^{cCA}± 1.41<br>(12.16±1.52)	64.89^{aA}±4.26<br>(+13.75±7.47)	64.82^{aA}±2.96<br>(+35.04±6.17)	57.53^{aA}±1.57<br>(+72.09±4.70)

ExGA: microencapsulated hop extract with Arabic gum; ExMD-GA: microencapsulated hop extract with mixture of maltodextrin and Arabic gum; ExMD: microencapsulated hop extract with maltodextrin; TPC: Total Phenolic Content (mg GAE g^{-1} dm); FRAP: Ferric Reducing Antioxidant Power (µmol Fe^{2+}Eq g^{-1} dm);TEAC: Trolox Equivalent Antioxidant Capacity (µmol g−1 dm). Data reported in parenthesis are loss percentages with respect to the initial content. Data on columns with different lowercase letters are statistically different at p level < 0.05. Data on rows with different capital letters are statistically different at p level < 0.05.
