# Supplementary figures and images for: Freeze-Drying Microencapsulation of Hop Extract: Effect of Carrier Composition on Physical, Techno-Functional, and Stability Properties
Source: Antioxidants (Basel). 2023 Feb 10;12(2):442. doi: 10.3390/antiox12020442 (PMC9951912; doi:10.3390/antiox12020442)

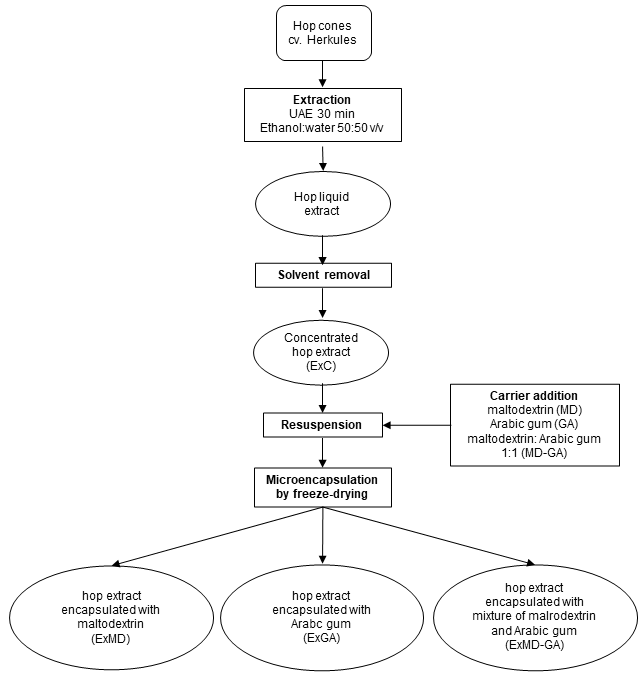

Supplement: Supplementary file 1 [file antioxidants-12-00442-s001.zip › Figure S1.tif]
